# Polymeric Nanoparticles for Mitochondria Targeting Mediated Robust Cancer Therapy

**DOI:** 10.3389/fbioe.2021.755727

**Published:** 2021-10-06

**Authors:** Yajing Sun, Qingshan Yang, Xue Xia, Xiaozhe Li, Weimin Ruan, Meng Zheng, Yan Zou, Bingyang Shi

**Affiliations:** ^1^ Henan-Macquarie University Joint Centre for Biomedical Innovation, School of Life Sciences, Henan University, Kaifeng, China; ^2^ Henan Key Laboratory of Brain Targeted Bio-nanomedicine, School of Life Sciences and School of Pharmacy, Henan University, Kaifeng, China; ^3^ Centre for Motor Neuron Disease Research, Department of Biomedical Sciences, Faculty of Medicine, Health and Human Sciences, Macquarie University, Sydney, NSW, Australia

**Keywords:** drug delivery, cancer therapy, mitochondria, polymers, nanoparticles

## Abstract

Despite all sorts of innovations in medical researches over the past decades, cancer remains a major threat to human health. Mitochondria are essential organelles in eukaryotic cells, and their dysfunctions contribute to numerous diseases including cancers. Mitochondria-targeted cancer therapy, which specifically delivers drugs into the mitochondria, is a promising strategy for enhancing anticancer treatment efficiency. However, owing to their special double-layered membrane system and highly negative potentials, mitochondria remain a challenging target for therapeutic agents to reach and access. Polymeric nanoparticles exceed in cancer therapy ascribed to their unique features including ideal biocompatibility, readily design and synthesis, as well as flexible ligand decoration. Significant efforts have been put forward to develop mitochondria-targeted polymeric nanoparticles. In this review, we focused on the smart design of polymeric nanosystems for mitochondria targeting and summarized the current applications in improving cancer therapy.

## Introduction

Cancer is a leading cause of morbidity and mortality worldwide (Sung et al., 2021). Faced with such threat, tremendous efforts have been dedicated in finding better and effective approaches to enhance therapeutic outcomes (Allahyari et al., 2017; Singh et al., 2018; Souza et al., 2019; Meric-Bernstam, 2021). Nevertheless, current strategies aiming to eradicate cancer and extend cancer patients survival have plateaued for most cases.

Mitochondria, known as a semi-autonomous organelle, exert crucial functions involved in energy production, cell differentiation, signal transmission, and apoptosis regulation (Jeong and Seol, 2008; Abate et al., 2020; Schirrmacher, 2020). In addition, mitochondria dysfunction is widely recognized to play a key role in tumorigenesis, and associated with multiple characteristics of cancer cells such as increased anabolism, uncontrollable replicative potential, insensitive to antitumor signals, and resisting organized cell death (Galluzzi et al., 2010; Księżakowska-Łakoma et al., 2014).

In the past 2 decades, mitochondria have become an attractive targeting site for anticancer treatment, and a wide collection of potential agents acting on mitochondria have emerged (Fulda et al., 2010; Lin et al., 2018; Huang et al., 2021). These agents generally target mitochondria functions, ultimately leading to cancer cell apoptosis via diverse mechanisms (Jeong and Seol, 2008). However, effective delivery of these agents specifically to mitochondria is yet faced with further challenges due to the additional phospholipid bilayer, their hydrophobic property and negative membrane potential (Smith et al., 2012). Thus, effective targeted delivery platforms towards the mitochondria are in urgent requirement.

Polymeric nanoparticles outstand in the design of smart targeted delivery that conquers the limitations discussed above, mainly ascribing to their outstanding features including ultimate biocompatibility, design-upon-request flexibility and synthesis (Chen, 2013; Begines et al., 2020). Specifically, surface modification of polymeric nanocarriers with lipophilic cations, peptides, or aptamers endows them with mitochondriotropic properties, giving rise to an attractive approach to design mitochondria-targeted therapeutics for cancer therapies (Pathak et al., 2015; Ahmed et al., 2021).

**SCHEME 1:**
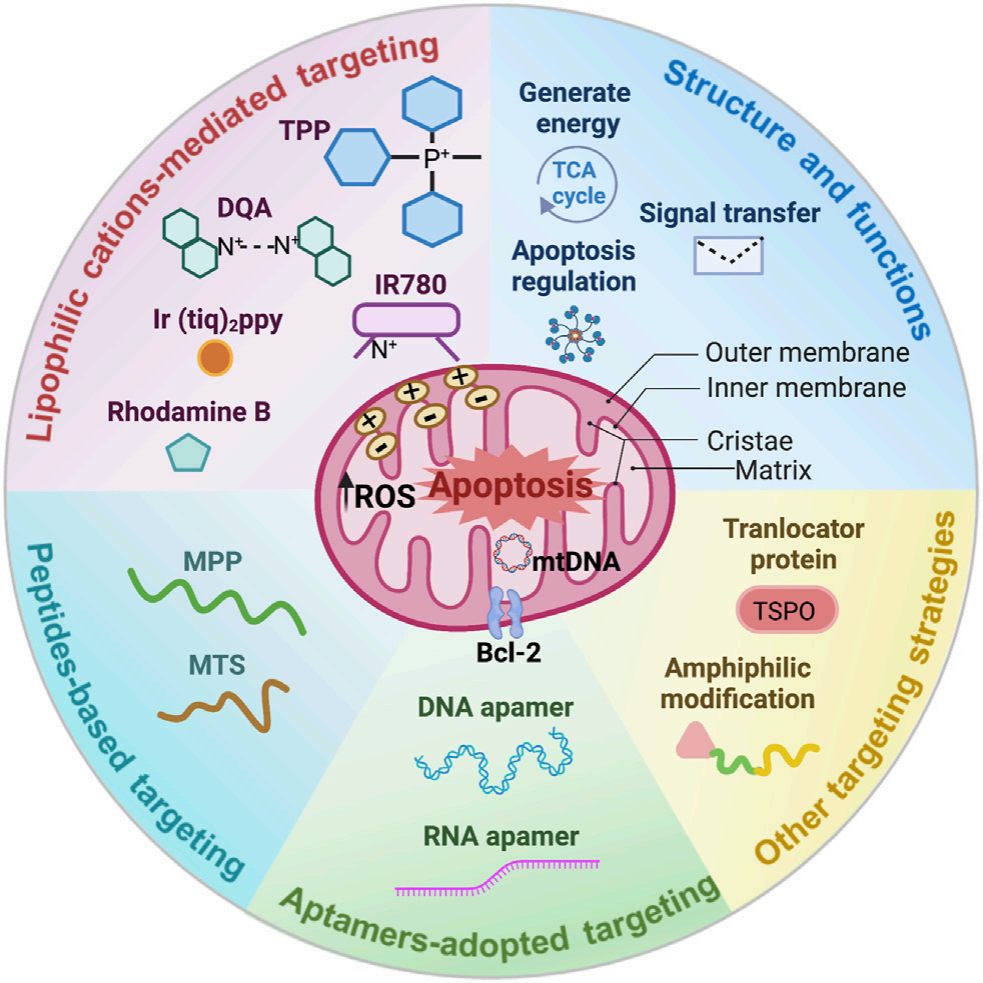
Unique structure and functions of the mitochondria, and outstanding designs for mitochondria targeting polymeric nanoparticles in cancer therapy.

This review will highlight the diverse strategies adopted in designs of mitochondria targeting polymeric nanocarriers for robust cancer therapies (Scheme 1), existing challenges and unexplored methods are also explored.

## Mitochondria and Cancers

Mitochondria are mainly composed of four compartments: the outer membrane, the inner membrane, cristae, and the matrix (Scheme 1) (Friedman and Nunnari, 2014). The inner membrane is highly folded to form the cristae, which provides enlarged surface area for adenosine triphosphate (ATP) synthesis but in the meantime, increased the difficulty for therapeutic agents accessing into the mitochondria matrix (Zick et al., 2009). These hurdles primarily resulted from the fact that the inner membrane not only hosts the majority of oxidative phosphorylation proteins including complex I (NADH dehydrogenase), complex II (succinate dehydrogenase), complex III (cytochrome bc1), complex IV (cytochrome c oxidase), and complex V (ATP synthase), but also processes a strong negative membrane potential of −160–180 mV required by the respiratory chain (Liberman et al., 1969).

In recent decades, it has been frequently proposed that mitochondria also play variety roles in cancer initiation, growth, and metastasis (Srinivasan et al., 2017), for which mitochondria continue to provide energy as a “power house” to satisfy the infinite proliferation of cancer cells, and contain special proteins that are important for aiding tumor cells to metastasize (Porporato et al., 2014). For example, it is reported that there is a strong correlation between the expression of transcription coactivator peroxisome proliferator-activated receptor gamma (PGC-1α) and the formation of metastases in invasive cancer cells (Lebleu et al., 2014). In malignant cancer cells, excessive levels of reactive oxygen species (ROS) are frequently reported and cause oxidative stress and mtDNA damage, resulting in mitochondria dysfunction (Boland et al., 2013; Rovini et al., 2021), further lead to tumor-promoting effects and cancer developments (Wei and Lee, 2003; Wallace, 2012). Therefore, mitochondria play a key role in the tumor cell proliferation and invasion (Kashatus et al., 2015).

Mitochondria have been proven to be effective targets in cancer therapies (Gogvadze et al., 2008), which can be achieved by targeting mitochondria, release therapeutic agents in mitochondria as triggered by the hyperthermia and ROS (Lopez and Tait, 2015). The essential roles of mitochondria played in intrinsic apoptosis pathway have been significantly recognized, and multiple pro-apoptotic and anti-apoptotic proteins held by mitochondria such as recombinant *p53* upregulated modulator of apoptosis (PUMA), B-cell lymphoma-2 (Bcl-2), Bcl-2 associated X protein (Bax), and so on, directly or indirectly modulating cancer cells death (Lu et al., 2016; Varela-López et al., 2021). Of noted, the multidrug resistance (MDR) is a great setback in cancer therapy (Vasan et al., 2019). Notably, the MDR considerably imputes to the overexpression of drug efflux pumps, which expend ATP supplied by mitochondria as well as pump drugs out of cancer cells in an energy-dependent way (Szakács et al., 2006; Guerra et al., 2017; García-Heredia and Carnero, 2020). A collection of recent findings emphasized that mitochondria are pivotal regulators in orchestrating immune antitumor responses through metabolic reprogramming of immune cells, eventually boosting immunotherapy effects (Araki et al., 2009; Wang et al., 2011; Bueno et al., 2021). Hence, it is a particularly prospective option to treat cancers via targeting and affecting mitochondria.

## Advantages of Polymeric Nanomedicines in Cancer Treatment

Growing numbers of clinical evidence proposed that current standard treatments for newly diagnosed cancer is surgical resection followed by radiotherapy and chemotherapy (Geifman and Butte, 2016; Chakrabarti et al., 2020). Unfortunately, the therapeutic efficiency is limited by the rapid clearance, MDR, and severe adverse effects of traditional chemical drugs (Norouzi et al., 2020). Development of novel drug delivery systems with minimal side effects while enhancing therapeutic efficiency is essential for successful future cancer therapy (Shi et al., 2017). By far, a collection of different types of nanocarriers have been developed. Among these, polymeric nanocarriers outshine with their unique features including supreme biocompatibility, design-to-order flexibility and synthesis, and readily surface decorations (Chen, 2013). These traits collectively offer an ultimate platform for developing polymer-based multifunctional nanomedicines. An assortment of various polymer-based nanocarriers have been designed to load and deliver different therapeutic agents in cancer therapies (Karabasz et al., 2020). These “smart” polymeric nanomedicines are capable of protecting the encapsulated drugs during blood circulation and releasing the drugs instantly under certain stimulus (Li et al., 2021), and a variety of targeting ligands could also be anchored on the surface of these polymeric nanoparticles to actively achieve specific targeted sites, resulting in robust cancer treatment with minimized side effects (Kamaly et al., 2012).

## Smart Designs of Polymeric Nanoparticles for Mitochondria-Targeted Cancer Therapy

As mentioned above, considering the crucial role of the mitochondria in various biological processes (Gogvadze et al., 2008; Fulda et al., 2010), specific targeting to cancer cell mitochondria could potentially be an alternative approach to trigger cell death for cancer treatment. For the purpose of delivering therapeutic agents into mitochondria, a series of factors need to be taken into consideration, such as the double and hydrophobic membrane system as well as their highly negative potential (Smith et al., 2012). In the meantime, directed from these unique properties of mitochondria, researchers take advantage of them as active targeting mechanisms for designing mitochondria-targeted nanocarriers. Surface engineering of polymeric nanoparticles with lipophilic cations, peptides, or aptamers can infuse them with mitochondriotropic properties, to exert ideal cancer therapeutic effects (Allemailem et al., 2021). In this section, we will summarize the various strategies adopted in the design of polymeric nanoparticles for mitochondria-targeted cancer therapy.

### The Lipophilic Cations-Mediated Mitochondria-Targeted Delivery

Most mitochondria targeting strategies utilize the highly hydrophobic and negative membrane potential of mitochondria inner membrane. For instance, it is reported that lipophilic cations are competent at penetrating the lipid bilayers and accumulating inside mitochondria (Zielonka et al., 2017). In account of this, numerous lipophilic cations, such as triphenylphosphine (TPP), dequalinium (DQA), heptamethine dye, cyclometalated iridium (III) complexes, and rhodamine derivatives, have been applied to polymeric nanocarriers for equipping them with mitochondria-targeted delivery (Battogtokh et al., 2018).

#### TPP-Based Mitochondria-Targeted Delivery

**FIGURE 1:**
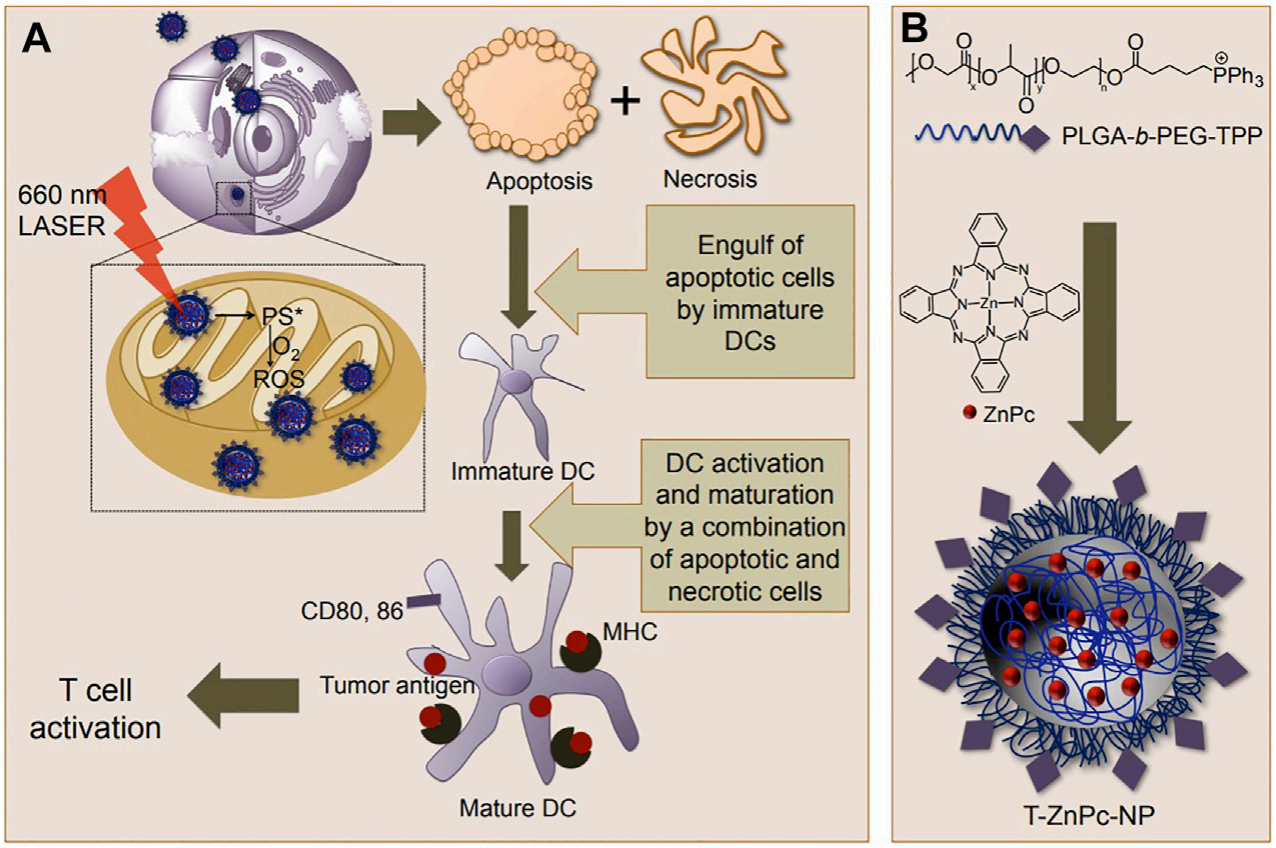
**(A)** Action of mitochondria-targeted nanoparticles (NPs) upon light activation inside mitochondria to produce ROS, which causes cell death and T-cell activation; **(B)** Synthesis of T-ZnPc-NPs using a nanoprecipitation method. Reproduced from ref (Marrache et al., 2013) with permission from Copyright 2013 American Chemical Society.

TPP is most frequently employed in decorating the surfaces of polymeric nanoparticles for mitochondria targeting, primarily due to its straightforward linking with different kinds of polymers (Guzman-Villanueva et al., 2019). Biswas et al. designed a mitochondria-targeted poly (amidoamine) (PAMAM) dendrimer modified with TPP (Biswas et al., 2012). Subcellular localization of the fluorescein isothiocyanate (FITC)-labeled dendrimers demonstrated great mitochondria targeting by these TPP-anchored nanoparticles, demonstrating the feasibility of and laying a solid fundamental for TPP-directed mitochondria targeting mediated cancer therapy. Similar results were also suggested by Wang et al., where another TPP-conjugated dendrimers delivery nanoplatform showed better mitochondria targeting and higher transfection efficacy than that of the non-modified dendrimers (Wang et al., 2014). In another work, Dhar et al. developed a smart engineered mitochondria-targeted polymeric nanoparticle system via blending poly (D, L-lactic-co-glycolic acid)-block-poly (ethylene glycol)-TPP (PLGA-PEG-TPP), with PLGA-PEG-OH or PLGA-COOH to generate an optimized size and surface charges nanocarrier for mitochondria-acting therapeutics delivery (Marrache and Dhar, 2012). Moreover, these polymeric nanoparticles could load small molecular therapeutics with high loading efficiency. The mitochondria uptake was confirmed by both qualitative and quantitative analysis of mitochondria fractions of cells incubated with the blended nanoparticles, showed that this TPP-engineered PLGA-based nanoparticle delivery system could enter mitochondria with high efficacy through fine-tuning their size and surface charges. Later, the same group also demonstrated the potency of mitochondria-targeted nanosystems based on PLGA-PEG-TPP to stimulate murine bone marrow derived dendritic cells (DCs) to secret interferon-gamma (IFN-γ) for cancer immunotherapy (Marrache et al., 2013). By using such modified nanocarrier to target mitochondria, a significantly increased level of interferon-gamma (IFN-γ) generated from cancer cells activated with mitochondria targeted immunotherapy. These TPP-endowed mitochondria-targeted polymeric nanoparticles can be applied in cancer treatments to provide robust improvements in therapeutic efficacy (Figure 1).

To further boost mitochondria-targeting delivery, Cho et al. linked two TPP molecules to the two ends of the poly (ε-caprolactone) (PCL) to form a bola-like amphiphilic TPP-PCL-TPP (TPCL) nanoparticle (Cho et al., 2015). Through the self-assembled method, TPCL nanoparticles can encapsulate both hydrophobic doxorubicin (Dox) and hydrophilic Dox (Dox·HCl). Most of these drug-loaded nanoparticles exert superb tumor-killing effects (about 7.5–18-fold) ascribing to their active mitochondria accumulation (2–7-fold). In order to obtain the best therapeutic effect whilst avoiding systemic side effects, polymeric nanocarriers have been designed to deliver drugs with controlled release properties. Various controllable release strategies, such as pH, redox potential (glutathione, GSH), enzyme, and ROS, have been used for targeted drug release (Tang et al., 2018). As mentioned above, mitochondria are the pivotal organelles for respiration, hence approximately up to 90% of cellular ROS generation occurs in these organelles (Tan et al., 2019). In view of this, ROS-responsive release could be complied in mitochondria. Yue et al. synthesized a mitochondria-targeting polymeric nanomedicine (Yue et al., 2016), which was prepared through thioketal linker-modified camptothecin (CPT) and TPP on PEG for delivery of the photosensitizer Zinc phthalocyanine (ZnPc). The thioketal linker is ROS-responsive. CPT and ZnPc are able to be quickly released under high levels of ROS in mitochondria. Their results showed efficient subcellular delivery into mitochondria and good ROS-responsive release of CPT and ZnPc. Also, upon irradiation, the released ZnPc triggered further ROS-induced cleavage of the thioketal linker. As such, it was found that ROS-responsive mitochondria-targeted nanoparticles could achieve a significant inhibition of lung cancer growth via ROS activated elevated chemo- and photodynamic therapy without marked adverse effects.

However, the highly positive charge of TPP-modified nanoparticles also results in quick blood clearance and thusly reduced accumulation at tumor sites. Moreover, these positive charged nanomedicines effortlessly bind to multiple biomacromolecules with negative charge during blood circulation, causing increased toxicity to normal cells (Guzman-Villanueva et al., 2019). Zhou et al. developed hybrid nanoparticles composed of poly (D,L-lactide-co-glycolide) (PLGA), TPP-containing polymer (C18-PEG-TPP) and a redox-responsive amphiphilic polymer (DLPE-S-S-PEG), in which the positive charges of TPP were shielded by the longer PEG chain, ensured prolonged blood circulation and reduced side effects (Zhou et al., 2017). After uptake by cancer cells, the mitochondria-targeting capacity was rapidly activated by the detachment of PEG layer under higher reductive conditions (1,000-fold of normal cells and blood), elevating the mitochondria localization and antitumor effects. Furthermore, compared with non-redox-responsive nanoparticles, these nanomedicines showed significantly higher mitochondria accumulation and superior therapeutic efficacy.

Enormous challenges caused by the MDR severely restrict the enhance of cancer therapeutic efficacy (Guerra et al., 2017). As we mentioned above, drug resistance significantly imputes to the overexpression of drug efflux pumps, which expend ATP supplied by mitochondria as well as pump drugs out of cancer cells in an energy-dependent way (Szakács et al., 2006; Guerra et al., 2017; García-Heredia and Carnero, 2020). Thus, targeting delivery of mitochondria-acting agents is an effective and promising strategy to overcome drug resistance to boost cancer therapy. Marrache et al. used a biocompatible polymeric nanoparticle based on PLGA-PEG-TPP to deliver cisplatin prodrug (Platin-M) specifically into mitochondria of cancer cells for overcoming resistance to cisplatin (Marrache et al., 2014). Platin-M inside the mitochondria is more active than cisplatin. The maximum Platin-M accumulation of this TPP-functionalized nanomedicine inside the mitochondria matrix attacked mtDNA and exhibited the most advanced sensitive to cisplatin in chemotherapy. Recently, Wang et al. synthesized a triblock copolymer (poly (ethylene oxide)-block-poly (propylene oxide)-block-poly (ethylene oxide), PEO-PPO-PEO, modified with TPP for the mitochondria-targeted delivery of paclitaxel (PTX) to combat drug resistance (Wang H. et al., 2020). To neutralize the positive charges of TPP, the hyaluronic acid (HA) with negative charges was grafted with this triblock copolymer via covalent bonds. The HA molecules could enhance the tumor targeting and specific uptake via CD44 molecule-mediated endocytosis as well as be degraded by hyaluronidase (HAase) in acidic lysosomes. Subsequently, these nanomicelles escaped from lysosomes and accumulated in mitochondria relying on the positive charged TPP. Finally, the tumor- and mitochondria-targeted nanomedicines activated intrinsic mitochondria apoptosis pathways by inhibiting antiapoptotic Bcl-2, leading to significant antitumor efficacy. Interestingly, in a drug-resistant breast cancer-bearing mouse model, the nanomicelles exhibited significantly reduced drug resistance via inducing the MDR cancer cell apoptosis.

Overall, TPP is the most commonly used as mitochondria-targeting ligand with minimal interference to the properties and functions of nanomaterials after linking to polymers. The TPP-decorated polymeric nanoparticles showed a promoted targeting ability to mitochondria and elevated drugs accumulation in mitochondria, resulting in superior tumor therapy as well as decreased drug resistance. Therefore, the TPP-mediated mitochondria-targeted strategy is significantly meaningful for improving cancer therapy.

#### Non-TPP Lipophilic Cations for Mitochondria Targeting

Other than TPP, there are a variety of lipophilic cations that have been extensively exploited as mitochondria-targeting ligands. DQA is a well-known lipophilic dication composed of two cationic moieties linked through a ten carbons alkyl chain (Song et al., 2015). This compound displays supreme potential in mitochondria targeting and antiproliferative activity in different cancer cell lines. In aqueous medium, DQA self-assembles and forms into liposome-like vesicles referred to as DQAsomes, which were applied for plasmid DNA (pDNA) delivery into mitochondria in earlier years (Bae et al., 2018). Shortly after, DQAsomes became widely used in the selective delivery of chemotherapeutic agents to mitochondria, and enhanced cancer cell death was observed (Zupančič et al., 2014). However, the terrible stability in high salt solution, the lack of exact mechanism and severe toxicity of DQAsomes limit their applications (D’Souza et al., 2003). Meanwhile, DQA has been investigated for its potential to be conjugated with polymeric nanoparticles (Weissig, 2015). Wang et al. built a functional Dox-loaded nanoparticle using folate-terminated polyrotaxanes along with DQA to overcome the MDR of cancers (Wang et al., 2015). Specifically, the folate-engineered nanoparticles can effectively pass the P-glycoprotein (P-gp), target and enter tumor cells based on the interaction between folate and the folate receptor on cancer cell membranes. Remarkably, the nanoparticles selectively located inside the mitochondria driven by DQA, ultimately leading to increased drug-resistant breast cancer cell death through the activated mitochondria apoptosis pathway and dropped resistance. By adopting a peptide linker, Mallick et al. constructed an amphiphilic polymer composed of glycol chitosan (GC) and DQA. DQA was selected for mitochondria targeting as well as function as the lipophilic section of polymer that was able to self-assemble into nanoparticles in aqueous solvent (Mallick et al., 2019). The GC component facilitated the cellular uptake and subsequent endosomal escape with no evident toxicity. The peptide linkage ensures the quick release of loaded drug (curcumin, CUR) under enzyme trigger. Compared to the controlled group, these DQA-functionalized polymeric nanomedicines exerted better cytotoxicity and therapeutic effects. Hence, DQA-based polymeric nanoparticles are suitable for mitochondria targeting drug delivery, and have the potential to treat drug-resistant cancers.

IR-780 iodide is a small molecule lipophilic cation heptamethine dye with the advantages of mitochondria targeting, near infrared fluorescence imaging, and photothermal and photodynamic activity (Conceição et al., 2013). The most outstanding limitations of the clinical application of IR-780 iodide are its poor solubility in physiological media and low dosage tolerance observed *in vivo* models (Conceição et al., 2013; Jiang et al., 2015). To tackle these barriers, IR-780 is conjugated with polymeric nanoparticles, these final products not only improve the aqueous solubility of IR-780 by 1,000-fold, but also reduce its side effects over 10 times (Jiang et al., 2015). Palao-Suay et al. conjugated IR-780 to PEG-poly [methacrylic derivative of α-tocopheryl succinate (α-TOS)] (MTOS) to generate self-assembled nanoparticles, which were suitable for photothermal and photodynamic therapies (PTT and PDT) (Palao-Suay et al., 2017). The results demonstrated that the multifunctional polymeric nanomedicines exhibited effective mitochondria targeting, severely quenched near infrared (NIR)-fluorescence, and much higher photothermal behavior. Enough internalization of IR780 dye in breast carcinoma cells treated with the modified nanoparticles, and subsequent NIR-laser irradiation dramatically resulted an imbalance of intracellular ROS and phototoxicity, which further induced cancer cells death. For instance, Liu et al. constructed a HA and IR825 conjugated polymeric nanosystem based on cholesterol-PEG (Chol-PEG) and encapsulated chemotherapeutics 10-hydroxycamptothecin (HCPT) into this nanocarrier (HA-IR825-Chol/HCPT) for combined chemo-photothermal therapy (Liu et al., 2020). It was found that HA-IR825-Chol/HCPT exhibited significantly enhanced accumulation in mitochondria, obviously upregulated apoptosis-related proteins, and demonstrated an amplified synergistic combination therapeutic efficacy, all of which were built on the basis of IR825-mediated mitochondria targeting.

Cyclometalated iridium (III) complexes are prominent anticancer drugs and photosensitizers for PDT for highly efficient ROS generation (approximately 100%) as well as a long triplet excited state lifetime (compared to tetraphenylporphyrin) (Huang et al., 2018). In recent years, cyclometalated iridium (III) complexes have been reported that they have a promising affinity for mitochondria, probably through similar mechanisms of lipophilic cations (Zhang et al., 2018). Lu et al. synthesized a cyclometalated iridium (III) complex, (Ir (tiq)_2_ppy), and covalently grafted it with polystyrene (PS)-PEG to prepare the Ir (tiq)_2_ppy NPs, which have water solubility and mitochondria-targeting capability (Lu et al., 2020). It showed that Ir (tiq)_2_ppy NPs were mainly accumulated in mitochondria of breast cancer cells, and were able to generate ROS under white light irradiation at low light intensity (5 mW cm^−2^). As a result, Ir (tiq)_2_ppy NPs showed a higher PDT efficiency than the group treated with other nanoparticles.

The rhodamine-based lipophilic cations are widely used as probes for the mitochondria membrane potential (ΔΨm) benefiting from their intrinsic fluorescence (Bhattarai et al., 2020). Rhodamine has a variety of structures, among which, rhodamine B (Rho B) is a red fluorescent probe with a great mitochondria-specific targeting ability (Wolfram et al., 2018; Wang X.-H. et al., 2020). Morimoto et al. developed a polymeric zwitterionic nanosphere for membrane translocation and organelle-selective delivery (Morimoto et al., 2016). The nanospheres were based on copolymers of 3-dimethyl (methacryloyloxyethyl) ammonium propanesulfonate and PEG methacrylate, p (DMAPS-PEGMA), internalized in cells through the plasma membrane. In particular, conjugation with Rho B empowered p (DMAPS-PEGMA) the ability to deliver selectively to mitochondria. As such, p (DMAPS-PEGMA)-Dox was shown to localize to both mitochondria and nucleus, effectively causing apoptosis in cancer cells.

### Active Mitochondria-Targeted Delivery Based on Peptides

Mitochondria-targeting peptides, a powerful substitution of lipophilic cations to actively sending drugs into the mitochondria, can be easily linked to polymeric nanocarriers and exhibit superb mitochondria-targeting ability. Recent studies have been carried out to demonstrate that multiple mitochondria-targeted peptides, such as mitochondria penetrating peptides (MPPs) and mitochondria-targeting sequence (MTS) peptides, are designed to penetrate the double lipid membrane as well as bind to the negatively charged inner mitochondria membrane via positively charged amino acids, accessing mitochondria and inducing cancer cell death (Constance and Lim, 2012).

MPPs are synthetic peptides that contain both cationic and hydrophobic residues. Given the special properties of these two components, MPPs are skilled at passing across the cell and mitochondria membranes (Jean et al., 2016). Selmin et al. developed a mitochondria-targeting hybrid polymer via functionalized PLGA with 6-mer MPP peptide for the first time (Selmin et al., 2017). The 6-mer MPP peptide is composed of alternating arginine and cyclohexylalanine, realizing successful entering the cells and mitochondria. These hybrid polymers could be obtained using different methods, such as solvent displacement and emulsification solvent evaporation. These authors also confirmed that the obtained nanoparticles had no cytotoxicity in methylthiazolyldiphenyl-tetrazolium bromide (MTT) assay, suggesting optimized biocompatibility. Soon afterwards, Cohen-Erez et al. designed amphiphilic and positive charged MPPs-combined nanoparticles (LND-mPoP-NPs) based on the anionic polypeptide poly gamma glutamic acid (γ-PGA) to deliver lonidamine (LND) into the mitochondria of breast cancer cells (Cohen-Erez et al., 2019). LND can inhibit glycolysis via inactivating the mitochondria outer membrane enzyme hexokinase, leading to the initiation of apoptosis. This study demonstrated that LND-mPoP-NPs exhibited a more reproducible tumor growth inhibition in comparison with LND in a xenograft breast cancer mice model empowered by the mitochondria targeting capability of MPPs.

MTS peptides, the protein peptides consisting of 20–40 amino acids, can be recognized by the surface receptors of mitochondria (Jean et al., 2016). Unlike other mitochondria targeting peptides, MTS peptides exhibit high specificity and precision to their corresponding mitochondria proteins (Lin et al., 2015). Moreover, MTS peptides can be processed by mitochondria processing proteinases, thusly releasing their delivered molecules (Lin et al., 2015). Therefore, these special features of MTS are adapted for the delivery of mitochondria-acting agents in cancer therapy with widely reported successes (Jean et al., 2016). However, several researches have suggested that naturally derived MTS peptides have lower efficacy for cell uptake compared to other mitochondria-targeting agents, presumably due to the large molecular size and poor solubility (Cohen-Erez et al., 2019). As such, it is of great importance to develop versatile polymeric nanosystems that are expected to complement the limitations of MTS peptides for specific mitochondria delivery.

### Adapting Aptamers to Achieve Active Mitochondria-Targeting

Active mitochondria-targeting employing aptamers has been a promising strategy for cancer treatments. In comparison to peptides, aptamers are easier to design and synthesize, and they are not immunogenic. Also, after further modification, aptamers are stable enough to resist biodegradation and denaturation (Bayat et al., 2018).

Given the normal bind between cytochrome c (Cyt c) and the inner mitochondria membrane via the anionic phospholipid cardiolipin, the nanoparticles modified with Cyt c aptamer have the potential to achieve mitochondria targeting (Mo et al., 2014; Chen et al., 2015). Chen et al. developed a smart ATP-responsive poly-L-lysine self-assembling nanoparticles for precise mitochondria-targeted cancer chemotherapy (Chen et al., 2017). In this system, a Cyt c aptamer was modified on the surface of the nanocarriers to enable these nanomedicines to high selectively accumulate in the mitochondria of cancer cells and promptly release the loaded Dox triggered by the high level of ATP in the organelle. The results revealed that the mitochondria-specific targeting nanomedicines distributed in the mitochondria (90.6%) were remarkably more than that in the lysosome, Golgi apparatus, and the nucleus, leading to promoted tumoricidal outcomes both *in vitro* and *in vivo*. Hence, the polymeric nanoparticles functioned by mitochondria targeting aptamers can deliver the drugs into mitochondria with precision, providing an opportunity to combat all mitochondria-associated diseases. However, there are still several drawbacks of aptamer modification design to achieve mitochondria targeting. For instance, the molecular weight of aptamers is relatively large, resulting in a low conjugation efficiency with nanocarriers as well as a change of the structure and properties of the nanomaterials (Odeh et al., 2019). Moreover, the relatively high cost of aptamers may ultimately impede the applications of aptamer-based mitochondria-targeted drugs delivery in cancer therapy.

### Other Strategies for Mitochondria Targeting

In addition to the approaches including lipophilic cations, peptides or aptamers for mitochondria-specific targeting, there are numerous other strategies for achieving mitochondria drug delivery in cancer treatments.

The translocator protein (18 kDa, TSPO), located on the outer mitochondria membrane, is a part of the cholesterol transport complex, responsible for transporting the cholesterol into mitochondria to synthesize steroids (Rupprecht et al., 2010). Moreover, TSPO is highly overexpressed in multiple diseases, including cancer, brain injury and inflammatory diseases, while minimally present in healthy tissues (Batarseh and Papadopoulos, 2010; Bhoola et al., 2018). Denora et al. developed a TSPO conjugated fourth generation dendrimer (G (4)-PAMAM) nanoparticle labeled with FITC for mitochondria targeting and imaging in glioblastoma (GBM) (Denora et al., 2013). To evaluate the ability of TSPO targeted nanoparticles to target mitochondria, they conducted subcellular fraction studies and co-localization assays in GBM (C6) cells. It is found that these nanoparticles clearly co-localized with mitochondria, evidencing the mitochondria-selective targeting of TSPO modified nanoparticles. In particular, TSPO is significantly upregulated in anti-inflammatory macrophages, providing a new way to promote mitochondria-related immunotherapy (Mukherjee et al., 2019). Sharma et al. adopted 5,7-dimethylpyrazolo [1,5-α] pyrimidin-3-ylacetamide (DPA), a novel class of TSPO ligands, to endow (G (4)-PAMAM) nanoparticles with mitochondria specific affinity property (Sharma et al., 2020). The DPA moieties decorated dendrimer nanoparticles presented promising targeting to GBM-associated macrophages (TAMs) and the mitochondria in TAMs. They also demonstrated that DPA-conjugated nanoparticles could co-localize with mitochondria in TAMs, stimulating excellent antitumor immune signaling in an orthotopic GBM model.

Interestingly, amphiphilic modification may be an effective approach for mitochondria-targeted delivery. Xi et al. reported a simple approach to deliver Dox to mitochondria in cancer therapy (Xi et al., 2018). The amphiphilic Dox was obtained by modified Dox with a lipophilic diacyl lipid (albumin protein) linked by PEG to enhance solubility. *In vivo*, the amphiphilic modification could reach and penetrate solid tumor by “hitchhiking” on albumin binding using albumin as an energy and nutrient source of tumor cells, and they could also locate at mitochondria after uptake by cancer cells. Although the mechanism by which amphiphilic modification targets mitochondria is yet elusive, the results showed that amphiphilic Dox effectively targeted and accumulated in the mitochondria, causing an obvious enhancement in oxidative stress in mitochondria, leading to significantly improved therapeutic effects. Thus, amphiphilic modification provides a novel strategy to make mitochondria location come true.

In recent years, it is reported that nanoparticles with smaller sizes (<10 nm) are able to target and penetrate into the mitochondria (Wongrakpanich et al., 2014). However, it is hard to control such a small size of polymeric nanoparticles with a relative large molecular weight of polymers. Therefore, design and develop new polymeric nanocarriers that are small enough is also a viable approach to optimize mitochondria targeting and accumulation.

## Conclusions and Perspectives

Mitochondria play a crucial role in a variety of physiological processes, hence mitochondria-targeting drug delivery is helpful and effective in cancer therapy. Unlike other subcellular organelles, the structure of mitochondria is unique and challenging for mitochondria-acting drug delivery. Furthermore, mitochondria-targeting strategies are also limited by the strong negative charged mitochondria inner membrane. To resolve these bottlenecks, an increasing number of strategies have been developed.

In this review, we emphasize the intelligent designs of commonly applied mitochondria targeting ligands decorated polymeric nanomedicines for enhancing cancer treatments. Lipophilic cations, peptides and aptamers are widely used mitochondria-targeting modifications in nanomedicines that can deliver various therapeutic agents to mitochondria. Polymeric nanoparticles are equipped with benefits in serving as mitochondria-targeted nanocarriers for their unique features including outstanding biocompatibility, flexible design and synthesis, and facile surface functionalization. Interestingly, the drug-loaded polymeric nanoparticles modified with these mitochondria targeting ligands exhibited a rapid and precise localization in mitochondria, leading to significantly higher cancer therapy effects. With the blessing of various controlled-release designs, the encapsulated drugs are quickly released and accumulated in the mitochondria, resulting in further promoted cancer treatment efficiency. Additionally, effective mitochondria-targeted cancer therapy is also promised to overcome drug resistance-caused ineffectiveness in treatment.

Nonetheless, it is still challenging to speed up future clinical applications of mitochondria nanomedicines for cancer therapies. First, the selective accumulation of these mitochondria-targeted moieties is largely given to the fact that their positive charges are adequately used. However, the rapid blood clearance and systemic side effects of these positive charged nanoparticles are a close second. Thus, enormous efforts should be devoted to developing new ligands that are able to target the powerhouse of cells with longer circulation and no undesired effects. In addition, there is a significant need to seek strategies to tackle the issue of mitochondria-targeting peptides being easily degraded by proteinases. By far, various surface modified polymeric nanoparticles that deliver drugs inside mitochondria have been clearly demonstrated with their ability to elevate tumor killing effects. While increased awareness should be called on the elegant design of multifunctional polymeric materials, which possess multiple properties (small size, appropriate charge, longer circulation, tumor, and mitochondria specificity) suitable for mitochondria-active drugs delivery. Accurate control of the physical and chemical properties of mitochondria targeted nanoparticles, including size distribution, surface charge, and the density of targeted molecules, is essential to achieve their clinical transformation. Additionally, the mitochondria targeting decorations are also easily applicable for other types of nanomaterials, such as liposomes and various inorganic particles, thusly it is promising to develop functional nanoparticles for elevating mitochondria-related therapies. Encouragingly, there has been great attention to mitochondria-targeted cancer therapies, and remarkable successes have been achieved. Drugs targeting delivery to other organelles other than mitochondria or simultaneous targeting multi-organelles may become the next hot issue in future cancer treatments.

In conclusion, polymeric mitochondria-targeting nanomedicines hold powerful promises for combating cancers. It is believed that mitochondria-targeted cancer therapies have the potential to eventually eliminate tumors with the development of the ultimate mitochondria-specific strategy and nanotechnology.
